# Tracking TRYCAT: A Critical Appraisal of Kynurenine Pathway Quantifications in Blood

**DOI:** 10.3389/fphar.2022.825948

**Published:** 2022-02-15

**Authors:** Violette Coppens, Robert Verkerk, Manuel Morrens

**Affiliations:** ^1^ Faculty of Medicine and Health Sciences, Collaborative Antwerp Psychiatric Research Institute (CAPRI), University of Antwerp, Antwerp, Belgium; ^2^ Scientific Initiative of Neuropsychiatric and Psychopharmacological Studies (SINAPS), University Psychiatric Centre Duffel, Duffel, Belgium; ^3^ Laboratory of Medical Biochemistry, University of Antwerp, Antwerp, Belgium

**Keywords:** psychiatry, kynurenine, tryptophan, depression, mood disorder, schizophrenia

## Introduction

**FIGURE 1:**
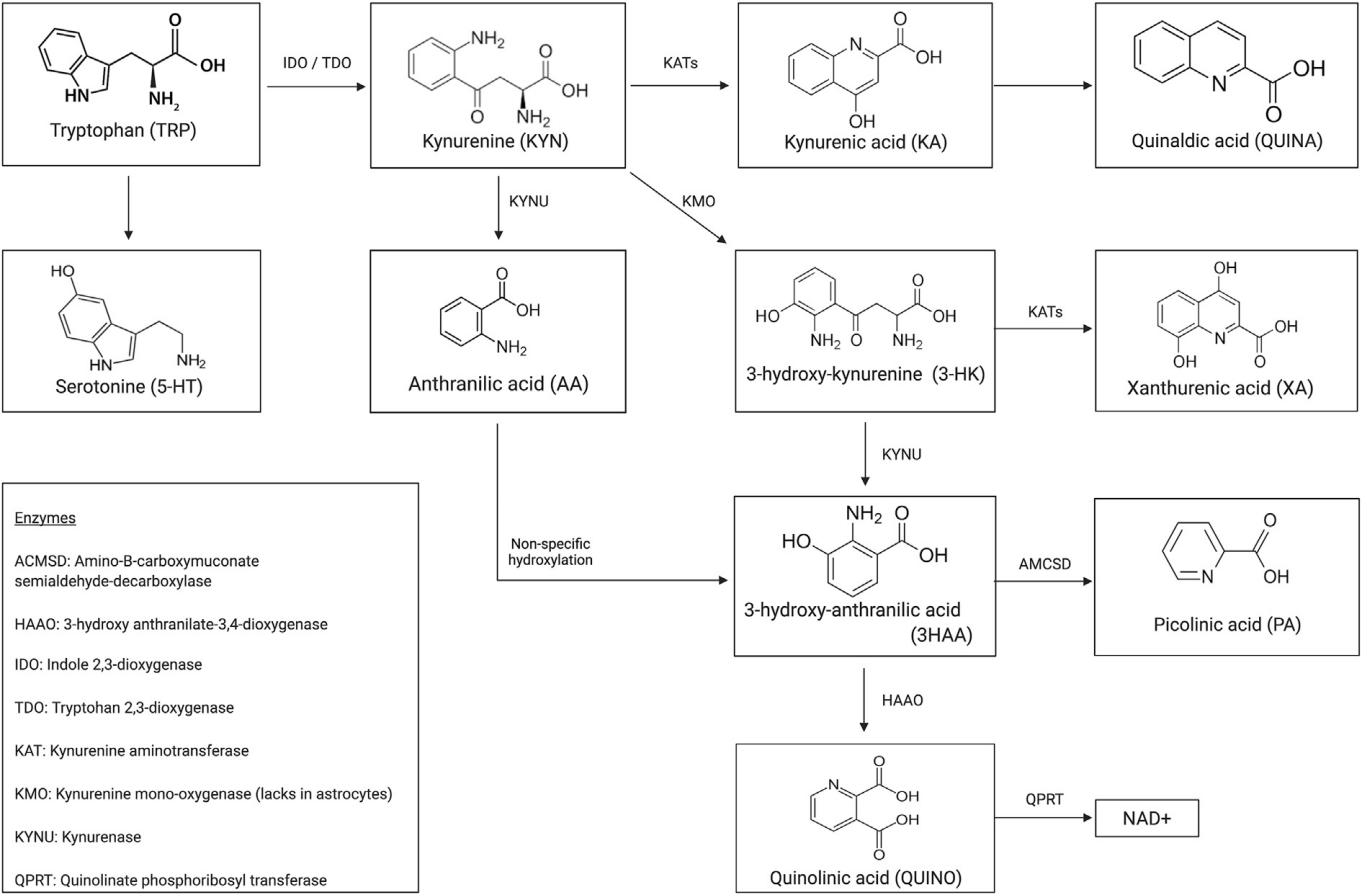
Schematic representation of the kynurenine pathway.

Immune dysregulation contributes extensively to the pathophysiology of multiple psychiatric illnesses (Coppens et al., 2019; Morrens et al., 2020a). Overall, 95% of the essential amino acid tryptophan (TRP) is degraded to kynurenine and either to its neurotoxic or neuroprotective immunogenic metabolites (see Figure 1 for a schematic illustration of the kynurenine pathway (KP)). A growing body of evidence testifies the neuromodulatory effects these microglia- or astrocyte-derived tryptophan catabolites (TRYCAT) have on the NMDA receptor. Hence, TRYCAT are hypothesized to link (systemic) immune responses to clinical symptomatology in psychotic and mood disorders.

A series of recent meta-analyses (Morrens et al., 2020b; Hebbrecht et al., 2021; Marx et al., 2021) confirm kynurenine pathway (KP) metabolite aberrances in these psychiatric illnesses in over 100 clinical studies. Unfortunately, the KP research field knows substantial though rarely contemplated pitfalls that warrant caution in the interpretation of findings. The current opinion piece will therefore zoom in on the conceptual validity of peripheral kynurenine metabolite quantification in psychiatric biomarker research. Furthermore, we will discuss the impact of sample heterogeneity, methodological reliability, and validity on study results. To conclude, the authors will put forward conceptual and methodological guidelines for future research into the kynurenine pathway and propositions for relevant future research avenues.

### The *Bloody* Brain Barrier

The bulk of literature on TRYCAT in psychiatry concerns quantifying metabolite levels in peripheral blood as a proxy measure for central inflammatory processes. However, as summarized in a recent systematic review by Skorobogatov et al. (2021), only TRP and KYN and to some extent 3-HK fluently travel over the blood–brain barrier (BBB). It remains undefined whether peripheral production of non-crossing metabolites such as kynurenic acid (KA) and quinolinic acid (QUIN) could indirectly reflect central inflammatory processes, for instance, through induction by BBB-crossing macrophages or macrophage-secreted cytokines. Nonetheless, for the metabolites traveling to the periphery over the BBB, extrapolation of the significance of KYN metabolization remains cumbersome. Illustratively, Skorobogatov et al. (2021) describe a strong correlation between peripheral and central KYN concentrations in two studies (Yuwiler et al., 1977; Haroon et al., 2020), while such interrelations remain absent for TRP itself. According to Yuwiler et al. (Yuwiler et al., 1977) and Curzon et al. (Curzon 1979), the observed CNS-periphery discrepancies in a mixed population of Huntington disease patients and healthy controls might be (partially) explained by the fact that most TRYCAT are known to bind circulating albumin (even with high affinity in case of TRP and KYN) and that only unbound (free) metabolites can enter the brain (Cangiano et al., 1999). This protein binding is advantageous as it provides a compound reserve in case of a temporal lack of supply. However, factors influencing the rate of albumin binding may intervene in a truthful peripheral representation of the central TRP metabolism. For instance, many drugs such as ibuprofen and valproate, pathology-induced non-esterified fatty acids (NEFAs), and other competing amino acids (tyrosine, phenylalanine, leucine, and valine) (Walker et al., 2019) can displace TRP from its binding site and in so doing directly affect central TRYCAT levels (Yang et al., 2014). Undoubtedly, this contributes substantially to TRYCAT aberrations found in medicated vs. unmedicated patients. Moreover, structural modifiers like glycation (Anguizola et al., 2016) or pH (Walser and Hill 1993) may cause conformational changes to the protein and thereby modify affinities. For detailed discussion, we refer to an excellent review by Feng Yang (Yang et al., 2014).

While their value as peripheral biomarkers for central neuroinflammatory processes remains questionable due to fickle BBB crossing and volatile albumin binding, peripheral TRYCAT levels may “retroactively” influence activity in the brain. *In exemplum*, decreased intracerebral KYN uptake following leucine competition for the active L-type amino acid transporter 1 (LAT1) inhibits depression-like behavior in mice (Walker et al., 2019). Additionally, the essential amino acid TRP inevitably needs to relocate from the periphery to the brain. Again, albumin binding plays a role as only unbound TRP and KYN can bind LAT1 to actively cross the BBB. Whether free or total TRP concentration is the major determinant for *in cerebro* bioavailability remains unelucidated. While centrally, KA is generated in brain astrocytes, skeletal muscle is its major peripheral source of production (Agudelo et al., 2014). As this metabolite is unable to cross the blood–brain barrier and the level of similarity/synchronicity between peripheral and central inflammatory processes is currently undetermined, its suggested anti-inflammatory neuroactive effects may not be directly extrapolatable from its peripheral concentration. At best, somatic metabolization of KYN to KA by kynurenine aminotransferases (KATs) results in lower levels of peripheral KYN available for import in the brain and subsequent central conversion to neurotoxic QUIN. This is indirectly evidenced by experiments with PGC-1a1 overexpression transgenic mice, which specifically upregulate KATs in skeletal muscle and subsequently show elevated blood concentrations of KA, lower (circulating KYN), and stress resistance (Agudelo et al., 2014). This may contribute to the decrease of symptoms observed in depressed patients following physical exercise training. In reverse, lower baseline KA levels in MDD and SZ patients could originate from decreased muscular production in poorly active patients. Higher levels of non-KA-conversed peripheral KYN increase *in cerebro* bioavailability and could as such induce depression-like behavior, as demonstrated in rodents (O’Connor et al., 2009).

### Living the TRYCAT Lifestyle

As illustrated above, muscle movement plays a major role in KA production. A sedentary lifestyle will therefore unquestionably affect kynurenine metabolization (Alme et al., 2021). Previous work showing that most KYN metabolites correlate with BMI (Zahed et al., 2021) indicates that lifestyle influences tryptophan metabolism also at the level of food intake. The amount and type of alimentation strongly define both the essential amino acid’s bioavailability and its metabolization. Vitamins B2 and B6 are KP cofactors; consequently, vitamin deficiency impacts kynurenine metabolite levels (Yeh and Brown 1977). TDO and IDO are iron porphyrin metalloproteins; hence, their enzymatic activity is dependent on iron supply. Moreover, prolonged inadequate food intake may result in low levels of serum albumin and as such interfere with free/bound metabolite fractions and consequent peripheral quantification (cfr. supra). As psychiatric patients are renowned for their poor eating habits, it is not inconceivable that varying peripheral TRYCAT levels largely reflect dietary insufficiencies. In support, Fellendorf et al. (Fellendorf et al., 2021) recently found TRP-to-KYN conversion in bipolar disorder to be facilitated by overweight and not by psychiatric symptomatology. This in turn raises the question as to whether TRYCAT aberrations in psychiatry are driven by syndrome-specific phenomena vs. being a by-product of other pan-pathological states like chronic stress-induced glucocorticoid resistance or sickness behavior hallmarking major depression and other psychiatric disorders (Zunszain et al., 2011).

According to Zahed et al. (Zahed et al., 2021), most TRYCAT remain relatively unaffected by smoking, which only appears to decrease QUIN, AA, and 3-HAA (Zahed et al., 2021). This may however be consequent to unassessed gender bias as Naz et al. (2019) demonstrate KYN and KA (but not TRP) levels to be lower in male smokers vs. non-smokers but remain unaffected in female smokers. Last, recreational drugs also interfere with homeostatic KP metabolite levels. Alcoholic beverages contain metabolically significant concentrations of KA, which is easily resorbed *via* the digestive tract and is quantifiable in peripheral blood. Hence, drinking beer and wine will artificially increase plasma [KA] (Turska et al., 2019). Cocaine use, on the other hand, will lower the amount of KA in blood (Araos et al., 2019). As comorbid substance use disorders are highly prevalent (∼30% (Toftdahl et al., 2016)) in mental health disorders, at least part of the variation in KP metabolite concentrations in psychiatry may be attributable to the (mis)use of varying types of recreational drugs.

Consult Gostner et al. (2020) for further elaboration on the interplay between lifestyle factors and TRP metabolization (Gostner et al., 2020).

### The Enzyme Enigma

Aberrations in KP metabolite levels have been largely attributed to deviant enzyme activity. However, the processing rate of these enzymes has rarely been directly assessed in psychiatric disorders. Instead, enzyme concentrations have mostly been approximated by gene expression analysis (Favennec et al., 2015) or by ratios of metabolite concentrations. Illustratively, the KYN/TRP ratio is suggested to reflect the TRP-to-KYN metabolizer IDO, and KAT activity is deemed deductible from [KA/KYN]. While peripheral metabolite ratios may reflect enzyme activities in closed systems such as cell cultures, assuming those correlations in blood, let alone in the brain, may stretch things too far. In support, non-correspondence of the KYN/TRP ratio with IDO activity was evidenced in patients with hemodialysis (Kato et al., 2010) and in children (Yarbrough et al., 2018). Again, albumin binding proves problematic as bound fractions do not reflect current production rates. Furthermore, several isoforms exist for most pathway enzymes. Does [KYN/TRP] reflect IDO1, IDO2, or TDO? Which of the two main KAT isozymes is represented by [KA/KYN]? Moreover, aberrations in renal clearance or in functionality of downstream enzymes may lead to upstream metabolite accumulation and will as such influence ratios independently of target enzyme activity. An elaborate critical appraisal of the KYN/TRP ratio as proxy for IDO activity can be found in the review by Abdulla et al. (Badawy and Guillemin 2019). Last, a recent review describes several alternative routes of KA synthesis *via* non-KATs and even enzyme-free mechanisms (Blanco Ayala et al., 2015; Ramos-Chávez et al., 2018). It is conceivable that similar alternative means of production also exist for other TRYCAT, which strongly dilutes the relevance of using metabolite ratios as a proxy for single (and mostly rather nonspecific) enzymes. As discrepancies between IDO1 mRNA and protein levels have been reported (Théate et al., 2015), this too proves to unreliably reflect KP enzyme activities and underscores the need for alternative quantification strategies.

### Tryptophan Catabolization: Not the Model Pathway!

Research on organs/diseases that are not or only hardly accessible/mimicable in humans often relies on animal studies to expand the pathophysiological knowledge. Nonetheless, caution is recommended when interpreting animal experimentation on TRYCAT in psychiatry. Not only are mood and psychotic disorders hard to model in animals, but the KP shows marked discrepancies in humans *versus* animals (Yeh and Brown 1977). Illustratively, TDO expression in humans is 5 to 10 fold lower than in rodents, and IDO activity suppresses human but not mouse lymphocyte proliferation (Torres Crigna et al., 2020). The latter reveals that these interspecies differences also functionally affect KP immunomodulation. Moreover, the fact that QUIN transport over the BBB occurs in gerbils but not in rats indicates that it even diverges between different species families of the same order (Heyes et al., 1997).

### Methodological Mayhem

KP research in the field of psychiatry is typically hallmarked by low sample sizes in heterogeneous study populations. Moreover, patient populations also differ from healthy controls regarding multiple demographic variables (BMI, smoking habits, *etc*.), which may in themselves impact TRYCAT levels. Specifically, all KYN metabolites increase with BMI and with age (except xanthurenic acid (XA) and 3-hydroxyanthranilic acid (3HAA)), and most are higher in males (Pertovaara et al., 2006; Zahed et al., 2021).

Alternate discrepancies in the literature may arise from the use of distinct analytical technologies. TRYCAT are almost exclusively measured using chromatographic methods such as high-pressure liquid chromatography (HPLC). These quantify total concentrations, while unbound concentrations may be more relevant (cfr. supra). Liquid chromatography–mass spectrometry (LCMS) and HPLC are performed with similar frequencies and show disagreement in concentration ranges, with some publications differing up to a staggering 1,000 times (Hartai et al., 2007; De Picker et al., 2019; Ulvik et al., 2020). Moreover, the concentration of several relevant kynurenine metabolites flirts with or even falls below the technological lower limit of detection of these methodologies (LC-MS quantification of QA, AA; unpublished data), implying caution concerning the scientific conclusiveness on their pathophysiological involvement. Last, sample collection is easily overlooked, though it is a crucial source of variance in TRYCAT quantification literature. Demonstrably, TRP is 15% higher in serum than in plasma (Yu et al., 2011) [with higher reproducibility in plasma, presumably due to coagulation-induced variance in serum (Bi et al., 2020)] and strongly increased in hemolytic samples (Kamlage et al., 2014). Heparin, an anticoagulant routinely used for plasma collection, is to be strictly avoided as it introduces severe chemical noise in LC-MS analyses (Yin et al., 2013) and interferes in albumin-TRP binding (Badawy 2010).

## Discussion

### What Path(way) to Take Next?

Tryptophan degradation through the kynurenine pathway has been widely implicated in the pathophysiology of a multitude of psychiatric disorders. KP derangement could even be involved causatively as mutations in SLC7A5, the gene coding for LAT1, have been linked to autism spectrum disorders (Tărlungeanu et al., 2016). Nonetheless, the research field is hallmarked by several pitfalls which might at least partially be circumvented by the hereby proposed guidelines.

→When quantifying peripheral metabolites to evaluate *in cerebro* bioavailability, unbound fractions in addition to total or bound levels need to be described. Thereby, one should take heed in optimizing their study design to account for modulators influencing freely circulating compound concentrations such as meal intake and type of collection tube (preferably collect fasting blood samples in EDTA-coated plasma tubes) (Badawy 2010). Alternatively, interference of albumin binding should be checked statistically by introducing [serum/plasma albumin] as a model covariate (van den Ameele et al., 2018; De Picker et al., 2019).
→When resorting to animal experimentation for fundamental exploration of kynurenine metabolization, larger mammalian species should be opted over rodent strains as KP characteristics in those animals show a higher degree of overlap with humans (Wang et al., 2018). Preferably, however, an in-depth scrutiny of the KP should be done *in situ* in human populations, for instance, *via* live imaging techniques (PET tracing of microglia activity) (De Picker et al., 2019), widespread characterization of KP metabolites in a multitude of bodily compartments (blood cells, urine, saliva, *etc*.), or clinical trials with compounds known to mediate the KP in animal research or other human pathologies such as IDO or KMO inhibitors (O’Connor et al., 2009; Prendergast et al., 2018; Réus et al., 2018).
→Metabolite ratios should be avoided to infer KP enzyme activity. Instead, enzyme concentrations or activities should be directly quantified in the fluidic compartments and/or judicious selections of blood. Successful assaying of KATs (in serum and erythrocytes), kynureninase (in lymphocytes), and IDO enzymes (in peripheral blood mononuclear cells) has been published elsewhere (Carlin et al., 1989; Ubbink et al., 1991; Hartai et al., 2007). Of note, immunogenic stimulation by for instance IFN-γ, is advised as basal enzyme activities may fall below detection limits in homeostatic circumstances (Edelstein et al., 1989). When direct measurement proves unattainable, enzyme activity can be inferred through detection of surrogate markers produced by high-specificity enzymes that respond to the same stimuli. Illustratively, neopterin, a pro-inflammatory marker of immune cell activation, proxies as a marker for IDO activity as IFN-γ also activates the key enzyme of neopterin synthesis (Werner-Felmayer et al., 1990). In parallel, enzyme activity can be deduced from direct measurement of protein levels. In support, *in vitro* IDO1 activity shows high correlation with protein expression (Wolf et al., 2009).
→In recent years, other pathway metabolites have gained interest as potentially having functional relevance in the pathophysiology of psychiatric disorders. Picolinic and xanthurenic acid, for instance, may act as trait markers for i.a. schizophrenia and therefore deserve to be more elaborately scrutinized in future endeavors (Fazio et al., 2015; Ryan et al., 2020).
→The recent emergence of sensitive commercial ELISA kits allows to demonopolize valuable TRYCAT metabolite and enzyme quantifications away from dedicated fully equipped, time-consuming, and expensive HPLC/LC-MS service labs toward broad-scale implementation to any research group with a basic laboratory infrastructure. As such, high-throughput quantifications on large sample sizes are now within reach.
